# Silver Vapor Supersonic Jets: Expansion Dynamics, Cluster Formation, and Film Deposition

**DOI:** 10.3390/ma16134876

**Published:** 2023-07-07

**Authors:** Alexander V. Bulgakov, Nikolay Y. Bykov, Alexey I. Safonov, Yuri G. Shukhov, Sergey V. Starinskiy

**Affiliations:** 1 HiLASE Centre, Institute of Physics of the Czech Academy of Sciences, Za Radnicí 828, 25241 Dolní Břežany, Czech Republic; 2 Center for Computer Engineering, Peter the Great St. Petersburg Polytechnic University, Polytechnicheskaya Str. 29, St. Petersburg 195251, Russia; nbykov2006@yandex.ru; 3 S.S. Kutateladze Institute of Thermophysics SB RAS, Lavrentyev Ave. 1, Novosibirsk 630090, Russia; safonoff82@mail.ru (A.I.S.); shukhovy@yandex.ru (Y.G.S.); starikhbz@mail.ru (S.V.S.); 4 Physics Department, Novosibirsk State University, Pirogova Str. 2, Novosibirsk 630090, Russia

**Keywords:** silver, supersonic vapor jets, nozzle expansion, atomic clusters, thin films, nucleation, metal nanostructures, mass spectrometry, direct-simulation Monte Carlo

## Abstract

Supersonic jets of metal vapors with carrier gas are promising for producing nanostructured metal films at relatively low source temperatures and high deposition rates. However, the effects of the carrier gas on the jet composition and expansion dynamics, as well as on film properties, remain virtually unexplored. In this work, the free-jet expansion of a mixture of silver vapor with helium in a rarefied regime at an initial temperature of 1373 K is investigated through mass spectrometry and direct-simulation Monte Carlo methods. Introducing the carrier gas into the source is found to result in a transition from a collisionless to a collision-dominated expansion regime and dramatic changes in the Ag jet, which becomes denser, faster, and more forward-directed. The changes are shown to be favorable for the formation of small Ag clusters and film deposition. At a fairly high helium flow, silver Ag_2_ dimers are observed in the jet, both in the experiment and the simulations, with a mole fraction reaching 0.1%. The terminal velocities of silver atoms and dimers are nearly identical, indicating that the clusters are likely formed due to the condensation of silver vapor in the expanding jet. A high potential of supersonic Ag-He jets for the deposition of nanostructured silver films is demonstrated. The deposited jet Ag_2_ dimers appear to serve as nucleation centers and, thus, allow for controlling the size of the produced surface nanostructures.

## 1. Introduction

Nanoparticles and nanostructured films play a key role in the development of modern electronics, photonics, and biomedicine. Metal clusters, due to their unique electronic, optical, and chemical properties [1], are mostly in demand for nanocatalysis, surface-enhanced Raman spectroscopy, sensor coatings, and nanodevice fabrication [2,3,4]. For medical applications, noble metal nanostructures are of particular interest. Pure and capped silver and gold particles are widely used for cancer treatment, biomedical imaging, drug delivery, diabetes management, and antibacterial material and drug supplements [5,6,7,8].

A large variety of physical and chemical methods exists for the synthesis of metal nanoparticles (see, e.g., reviews [9,10,11,12]). For the fabrication of noble metal nanoparticles, the most popular techniques include diversified methods of chemical and electrochemical reduction [12,13,14], evaporation–condensation in a carrier gas [12,15], laser ablation in both gas environments [4,16,17] and liquids [16,18], magnetron sputtering [9,19], discharge plasma [9,20], and vacuum gas jet deposition (VGJD) [21,22]. Every method has its advantages and disadvantages. Chemical methods allow the mass production of metal nanoparticles with uniform size distributions, but the drawback is the inherent solvent contamination and the necessity to use surfactants or ligands to stabilize the nanoparticles [13,14]. Using the evaporation–condensation technique, pure aerosol particles can be produced in large quantities, but the particle size distribution is usually broad, which is inappropriate for most applications, and additional particle separation techniques are required [15] that considerably reduce the method’s efficiency. Methods based on laser ablation and sputtering enable the fabrication of contamination-free nanoparticles with the possibility to control the nanoparticle size, but the productivity of these methods is rather low, which prevents their large-scale applications. Recently, a technique was developed to considerably increase the productivity of nanoparticle synthesis by laser ablation in liquids up to a g/h level [23], but it was based on an expensive, troublesome scanning system that limited its practical usage. In this respect, the vacuum gas jet deposition (VGJD) method with its high yield of pure nanoparticles appears to be particularly promising.

The VGJD method involves the heating and evaporation of metal inside a crucible, the mixing of metal atoms with an inert carrier gas in the crucible volume, and an outflow of the hot gas mixture through a nozzle to form a supersonic jet expanding into a vacuum or a rarefied environment. The produced metal particle flow is then deposited onto a substrate surface where nanostructure formation and growth occur. The method is particularly attractive for large-scale synthesis since it allows high-rate deposition onto large-area substrates with the possibility of fine-tuning the nanostructure size. The VGJD fabrication of nanostructured silver films with excellent uniformity over a ~100 cm^2^ area was recently demonstrated [22], which does not seem to be achievable using other methods. Various processes are involved in the VGJD method, including evaporation of a metal into a carrier gas, acceleration and cooling down of the metal vapor during supersonic expansion, formation of metal clusters in the supersonic jet, and, finally, deposition of the jet species onto a substrate and formation of nanoparticles. The interplay of the involved processes is complicated and still poorly understood, and the deposition conditions are usually obtained empirically by the trial-and-error method. The melt temperature inside the crucible, load parameters of the carrier gas, nozzle geometry, residual pressure in the vacuum chamber, material, and temperature of the substrate affect the properties and morphology of the produced nanostructured film. Important and little-investigated features of the VGJD method are the possible presence of small metal clusters in the deposited flow and their role in the film growth process.

The VGJD method is, in fact, a modification of the well-developed nozzle cluster beam deposition (CBD) method, where metal clusters are generated in supersonic nozzle expansion [24,25]. To produce clusters using the CBD method, a quite large vapor pressure of the order of 10 kPa in the crucible volume is needed [24,25,26]. For relatively high-melting metals of interest for VGJD (e.g., Ag, Au, Al, and Cu), such a pressure is achieved at fairly high crucible temperatures of around 2000 K or higher. This can result in contamination of the produced clusters and supported nanostructures by source material atoms, which is unacceptable for some applications. The VGJD method operates at considerably lower crucible temperatures (e.g., in the range of 1200–1600 K for silver nanoparticles) with typical vapor pressures 2–3 orders of magnitude lower than those used in the CBD method [21,22], which makes this technique much more practical.

There has been some controversy regarding the clustering of metals like silver and aluminum in vapor nozzle expansions [21,24,27], and a rigorous quantitative description of the clustering process is lacking. Hagena demonstrated a similarity between rare gas and metal vapor expansions, and the developed scaling law seems to predict qualitatively the metal condensation onset [28]. According to this law (although it is not directly applicable to the expansion of a metal vapor-gas mixture) and to our simulations of silver jets [29], one cannot expect the formation of small metal clusters (dimers or trimers) in a VGJD jet at source temperatures below 1600 K. However, Ag_2_ and Ag_3_ clusters were recently registered under these conditions at a fairly high carrier gas flow [22]. Obviously, Hagena’s clustering criterion should be revisited for supersonic jets of metal vapors mixed with a considerable excess of carrier gas. Small metal clusters, if they are present in the vapor flow, can strongly affect the film growth since the deposited clusters can serve as nuclei in nanostructure formation at the surface [22,30]. Therefore, the VGJD method enables the formation of fairly large nanoparticles without heating the surface [22], which opens the possibility of using low-melt substrate materials, like plastics. It is notable that another similar technique for metal deposition, molecular beam epitaxy (MBE), operates at much lower vapor pressures (typically 0.01–0.1 Pa [9]) where the expansion is collisionless and no clusters are formed.

The type of carrier gas is a key factor for the optimal operation of a VGJD source. Two gases, argon and helium, are typically used as carriers for the generation of gas-phase clusters in the above-mentioned physical methods (laser ablation, sputtering, and nozzle expansion). Argon is less expensive, is more suitable for sputtering processes, and can promote clustering onset [31]. However, gas-phase condensation and metal cluster formation during supersonic expansion proceed more effectively in lighter helium [19]. Additionally, clusters formed in helium are found to be colder than those generated in argon under identical expansion conditions [32], which is favorable for cluster growth. Furthermore, metal particles are accelerated to higher velocities in helium than in argon, and thus, they have higher surface mobility when deposited on a substrate [33], which is important for the formation of supported nanoparticles. Finally, in fairly dense argon jets, condensation of the carrier gas itself with the formation of mixed gas-vapor clusters can occur [31], a process that is desirable to avoid and which does not exist in helium jets. Therefore, helium is considered a more appropriate carrier gas for the VGJD technique.

In this work, we perform experimental and theoretical studies of processes involved in the VGJD method for the case of silver deposition in helium carrier gas with the main focus on small silver cluster formation in an Ag-He jet and their influence on the produced film morphology. The experiments are performed using a gas-dynamic sampling scheme of the beam from an expanding jet, and the compositions and velocities of the beam particles are analyzed using time-of-flight mass spectrometry. The VGJD conditions resulting in small silver cluster (dimer and trimer) formation in a jet are found for the first time. The velocities of the deposited atoms and clusters are measured using a technique developed previously for laser ablation experiments [34,35]. Film deposition for cases of the presence and absence of silver clusters in the jet is performed, and a clear difference in the film morphologies for these two VGJD regimes is demonstrated. A theoretical analysis of an Ag-He jet under the considered conditions is performed using the direct-simulation Monte Carlo (DSMC) method [36,37]. This technique allows simulations of spatial flows of a complex geometry, taking into account clustering processes. The DSMC method has previously been successfully employed for aerospace applications [38,39], solutions of astrophysical problems [40], and problems of material science [41].

The purpose of this paper is, thus, manifold and includes (i) analysis using both experiments and modeling of the influence of basic VGJD parameters on the characteristic of the deposited flow, (ii) finding conditions for silver dimer formation in the expanding rarefied jet of the Ag-He mixture, (iii) analysis of the structure and morphology of the obtained nanostructured films of different VGJD regimes, and (iv) revealing the most appropriate parameters for silver clustering models based on a comparison of the experimental and simulation results.

## 2. Experimental Methods

**Figure 1:**
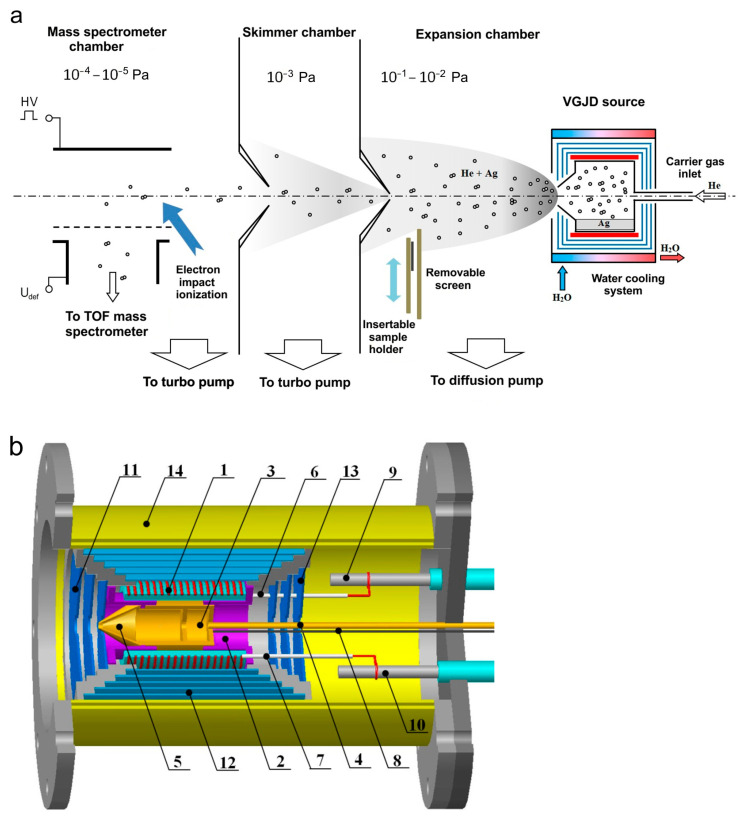
(**a**) Schematic of mass spectrometric measurements and deposition experiments (adapted with permission from Ref. [22]). An atomic (cluster) beam was sampled from the supersonic Ag-He jet using a conical skimmer and collimator. (**b**) VGJD source: 1—heater, 2—locating block, 3—crucible, 4—gas inlet channel, 5—nozzle, 6 and 7—quartz tubes, 8—thermocouples, 9 and 10—electrical feedthroughs, 11–13—radiation shields, 14—water-cooled housing.

The generation of a supersonic vapor jet of an Ag-He mixture, investigation of the jet, and deposition of the jet species on substrates were performed in a vacuum gas jet deposition (VGJD) chamber equipped with a mass spectrometry (MS) apparatus for the investigation of gas/plasma flows [17,29,42]. A general scheme of the experimental setup is shown in Figure 1a. A silver vapor was produced in a crucible inside a VGJD source, where the vapor was mixed with helium carrier gas and expanded through a sonic nozzle (orifice) into a vacuum chamber (base pressure of 10^−6^ mbar) to form a supersonic jet. At a distance of 88 mm from the nozzle exit, the jet was skimmed with a conical sampler (orifice diameter of 7 mm) to form a particle beam that passed through a separately pumped vacuum chamber, was collimated with a second skimmer, and finally entered the high-vacuum chamber (base pressure of 10^−7^ mbar) of a home-made reflectron-type time-of-flight mass spectrometer (TOF MS). At a distance of 687 mm from the first skimmer, the beam particles were pulse-ionized with 90 eV electrons (pulse duration of 1 µs), and the produced ions were pushed out using a high-voltage pulse perpendicular to their original flight direction for TOF MS analysis of the masses and velocities of the particles. Details of the mass spectrometric measurements are described elsewhere [22,35,42,43].

Figure 1b shows the VGJD source in more detail. A cylindrical molybdenum crucible (3) on a stainless-steel base (2) was located inside a quartz tube equipped with an external resistive heater (1). The crucible was loaded with approximately 1.5 g of silver metal (99.99% purity, Sberbank, Moscow, Russia). The crucible ended with a convergent conical nozzle (5) with a 3 mm diameter exit orifice. In all the experiments reported here, the temperature inside the crucible was fixed at 1373 K (i.e., well above the silver melting point of 1235 K) and was monitored using thermocouples (8). The corresponding saturation silver vapor pressure was 5 Pa [44]. The source had several radiation shields (11–13) to reduce heat losses. Using a gas inlet channel (4), inert carrier gas (helium, 99.999% purity, NII-KM, Moscow, Russia) was introduced to the source above the silver melt, where it was mixed with the Ag vapor and finally expanded through the exit orifice as a vapor-gas Ag-He mixture supersonic jet. The helium flow rate was varied in the range of 0–500 standard cubic centimeters per minute (sccm) using flow controllers (RRG12, Eltochpribor, Zelinograd, Russia). The total stagnation pressure in the source was also monitored indirectly in a tube connected to the gas inlet channel using a Barocel 600 capacitor gauge (Edwards, Burgess Hill, UK). In the studied range of the gas flow rate, the measured pressure varied in the range of 0.01–12 kPa. However, this value was obviously different than the actual mixture pressure in the source, particularly due to different temperatures. Therefore, the measured pressure values were considered as indicative ones and were not used in our simulations. The experimental setup was equipped with high-speed vacuum pumps (10,000 L/s diffusion pump for the expansion chamber and 1500 and 1000 L/s turbo pumps for the skimmer and MS chambers, respectively (Figure 1a)) that allowed us to maintain the required high vacuum in the MS chamber under high helium gas loads. The source temperature reached its steady-state value of 1373 K approximately 30 min after the heating started and was kept at this level with a precision of 1 K by maintaining the heating current using an ELIM EL-SS3000-108 power supply. A silver load of 1.5 g was sufficient for 5–10 h of the experiments, depending on the helium flow, which strongly affected the silver flux at a fixed source temperature (see Section 4.2).

The velocities of the jet species were measured using a mass spectrometric method developed previously for orthogonal recoil geometry [34,35]. The method was based on the fact that the ions, being pushed out perpendicularly to their flight for the MS analysis, kept their original jet velocity. Therefore, to reach a distant MS detector, the ions had to be properly deflected in an external electrical field to compensate for the axial ion drift. In our setup, this was realized with a pair of deflecting plates (Figure 1a) with the applied voltage $U_{\mathrm{def}}$ connected to the mean particle velocity u through the relation $U_{\mathrm{def}}=Cum^{1/2}$, where m is the particle mass and C is a constant depending on the MS geometry and settings. Here, to measure the velocity, the voltage $U_{\mathrm{def}}$ was optimized for every expansion condition and every jet particle (atoms and clusters) to maximize the MS signal. The C value was determined in separate calibration experiments by detecting In^+^ ions (mass of 115 u close to that of Ag atoms) that were laser-desorbed from an InP semiconductor target (a 0.5 mm thick wafer) under vacuum conditions when the ion velocity could be precisely measured using their time-of-flight [35,42]. The uncertainty in the velocity determination was about 10% for the lowest u values (without helium) and decreased when the Ag species were accelerated by the carrier gas.

In addition to the mass spectrometric characterization of the Ag-He jet, we also deposited thin silver films from our VGJD source. The deposition was performed in the same expansion chamber by introducing silicon or quartz substrates to the jet axis perpendicular to the flow at 310 mm from the nozzle exit (Figure 1a) using a translation stage. A removable screen was installed in front of the substrate to open it for deposition during a fixed period of time and, thus, to control the film thickness. The substrate temperature was monitored with a thermocouple, and although the substrate was not heated on purpose, it increased during the deposition by 20–30 K above room temperature due to radiation from the hot VGJD source. The morphology and thickness of the produced films were analyzed as functions of the deposition conditions using scanning electron microscopy (SEM, JEOL JSM-6700F, JEOL, Tokyo, Japan). In addition, the mass-equivalent thickness of the films was determined in optical transmittance measurements [22,45] using a SF-2000 UV/Vis spectrophotometer (OKB Spectr, St. Petersburg, Russia) at a 200 nm wavelength.

## 3. Simulation Details

### 3.1. Problem Formulation

**Figure 2:**
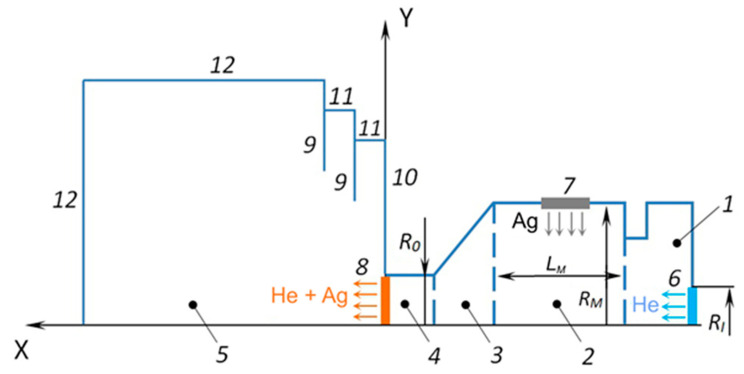
Scheme of the simulation region: 1—initial internal cylindrical section, 2—main internal cylindrical section with the Ag melt, 3—converged cone section, 4—cylindrical nozzle section, 5—expansion region, 6—He inlet, 7—Ag evaporation surface, 8—nozzle exit, 9—thermal shields, 10 and 11—side walls of the shields, 12—boundaries of the expansion region.

In these simulations, the problem was considered in the axially symmetric form with longitudinal X and transverse Y coordinates. The scheme of the simulation region is presented in Figure 2. The source/crucible consisted of two internal cylinders (1, 2), a converging conical part (3), and a cylindrical exit nozzle unit (4). The radius and length of the main cylindrical part (2) of the source (which contained the Ag melt) were *R_M_* = 9.5 mm and *L_M_* = 22 mm, respectively. The source had an inlet orifice (6) for introducing the carrier gas (helium) with a radius of *R_I_* = 2.5 mm. The radius of the source outlet orifice (8) was *R*_0_ = 1.5 mm. All these dimensions corresponded to the geometry of the real VGJD source used in the experiments. An important simplification of the present model as compared to the experimental situation was the substitution of the flat surface of the Ag melt inside the source with an evaporating ring of the same area. This approach allowed for reducing the real 3D flow of the vaporizing Ag atoms inside the crucible to an axially symmetric flow and considerably accelerated the computation process. The ring was located at the center of the main source cylinder. The expansion region (5) was limited by the boundaries (11, 12) and contained thermal shields (9).

The fluxes of Ag atoms through the evaporating surface (7) and the flux of He atoms through the inlet surface (6) were simulated using the following equation [36]:

(1) $$F^{+}=n{\left(\frac{kT_{0}}{2\pi m}\right)}^{1/2}\left[\exp\left(-s^{2}\right)+\pi^{1/2}s\left(1+\mathrm{erf}\left(s\right)\right)\right]$$

where $T_{0}$ is the source temperature, n is the concentration, m is the mass of particles, $s=u/{\left(2kT_{0}/m\right)}^{1/2}$ is the velocity ratio (*u* is the mean particle flow velocity), erf is the error function, and *k* is the Boltzmann constant. The velocity distribution for particles introduced into the computational domain corresponded to the Maxwellian one.

For Ag atoms evaporating through the surface (7), Equation (1) for the flux $F_{s,\mathrm{Ag}}^{+}$ corresponded to the Hertz–Knudsen equation with the parameters $s_{\mathrm{Ag}}=u_{\mathrm{Ag}}=0$ and $n=n_{0,\mathrm{Ag}}=p_{0,\mathrm{Ag}}\left(T_{0}\right)/kT_{0}$, where $n_{0,\mathrm{Ag}}$ and $p_{0,\mathrm{Ag}}$ are the atom concentration and the pressure of saturated Ag vapor evaluated in [44], respectively. Silver atoms scattered back to the evaporating surface were condensed at this surface and excluded from the simulations. The Ag atom flux and corresponding energy flux through the helium inlet orifice was assumed to be zero, and the specular reflection condition was set at this surface. It was assumed that Ag atoms reflected diffusively from other surfaces inside the source, with the total energy accommodation at the temperature $T_{0}$. The atoms incident on the thermal shields condensed at the surface and were excluded from the simulations.

The total flux of He atoms through the surface (6) was defined by the experimental value of the carrier gas flow rate $Q_{\mathrm{He}}$. The value of the He inlet flow velocity was unknown, and it was assumed that $s_{\mathrm{He}}$ = 0.1 at the surface (6). The concentration $n_{\mathrm{He}}$ was then defined from Equation (1). Specular reflection for the helium atoms backscattered to the surface (6) was assumed, whereas they reflected diffusively from other internal surfaces and thermal shields with the total energy accommodation. Both He and Ag atoms crossing the external boundaries of an expansion region (10, 11, and 12) were excluded from the calculations. The source temperature $T_{0}$ was fixed, as in the experiment, at the value of 1373 K.

Therefore, according to the present model, the characteristics of the expanding Ag-He jet were determined with the following parameters: source temperature, helium flow rate, source geometry, shield temperature, particle collision parameters, and chemical reaction models.

The simulations were performed using home-made parallelized DSMC software [46]. A no-time counter (NTC) collision scheme [37] was employed for collision process modeling. A zone mesh matched with the area boundaries was employed for the simulations. Depending on the rarefaction degree, the number of cells was in the range of 10^4^–5 × 10^4^. The size of cells was approximately equal to or less than the local mean free path of atoms in the corresponding zones, according to the requirements of the DSMC method [36,37]. The time step was 1.5 × 10^−9^ s, which was less than the local mean time between particle collisions in any flow point for any degree of rarefaction. The number of simulated particles was in the range of 10^8^–2 × 10^8^.

The simulations in the above problem formulation were rather time-consuming. The main computational costs were associated with the calculations of the flow inside the source, especially for cases when we focused on the investigation of silver Ag_2_ dimers (see Section 3.2) whose fraction in the flow was quite low. Therefore, additional simulations were performed for a simplified, truncated geometry of the source to determine the parameters of the dimers. In this case, only the cylinder (4) and expansion zone (5) were considered. The boundary conditions in the inlet boundary (i.e., the boundary between cylinders (3) and (4)) were set in a selection process where the parameters of Equation (1) were chosen in such a way that the species fluxes through the source outlet and the parameters of Ag and He in the inlet section reproduced the corresponding parameters obtained in the complete geometry simulations.

### 3.2. DSMC Collision Models

For the description of collisions between the jet particles, a variable hard-sphere (VHS) model [36] was used in this work. According to the VHS model, the collision cross-section diameter d is a function of the relative velocity of colliding particles *c*:

(2) $$d=d_{ref}{\left(\frac{c_{ref}}{c}\right)}^{\omega -1/2}=d_{ref}{\left[{\left(\frac{2kT_{ref}}{\mu c^{2}}\right)}^{\omega -1/2}\frac{1}{\Gamma \left(5/2-\omega \right)}\right]}^{1/2}$$

where $d_{ref}$ and $c_{ref}$ are the corresponding values at a reference temperature $T_{ref}$, *ω* is a parameter characterizing the temperature dependence of the gas viscosity (viscosity index), *µ* is the reduced mass of the colliding atoms, and Γ is the Gamma function. For helium atoms, $d_{ref,\mathrm{He}}$ = 0.233 nm at $T_{ref}$ = 273 K, and *ω*_He_ = 0.66 [36]. In contrast to noble gas atoms, reliable data on the VHS parameters of metal vapors are absent, as their determination is nontrivial [47]. Here, we assumed hard-sphere (HS) mechanics for Ag–Ag collisions, with $d_{ref,\mathrm{Ag}}$ = 0.29 nm and *ω*_Ag_ = 0.5 [48]. It should be noted that the HS model has previously been successfully employed for modeling rarefied gas flows using the DSMC approach [49,50]. The heteroatomic Ag–He collisions were considered within the VHS model, with the $d_{ref}$ and *ω* parameters taken as the mean values of the corresponding parameters for the Ag–Ag and He–He collisions [36,37].

In the simulations, we considered the formation of Ag_2_ dimers in the Ag-He mixture flow in triple collisions (forward process) and their disintegration through collisions with Ag and He atoms (reverse process) according to the following reactions [36,51]:

Ag + Ag + Ag → Ag_2_ + Ag, (3)

Ag + Ag + He → Ag_2_ + He, (4)

Ag_2_ + Ag → Ag + Ag + Ag, (5)

Ag_2_ + He → Ag + Ag + He, (6)

with the temperature-dependent rate coefficients of forward (Equations (3) and (4)) and reverse (Equations (5) and (6)) reactions being *K*_12,Ag_, *K*_12,He_, *K*_21,Ag_, and *K*_21,He_, respectively.

The diameter of Ag_2_ dimers was estimated using the spherical drop model as $d_{2}=2r_{w}g^{1/3}$ = 0.42 nm with cluster sizes of g = 2 and $r_{w}$ = 1.66 Å [52]. Correspondingly, the $d_{ref}$ diameter in the VHS model for Ag–Ag_2_ and He–Ag_2_ collisions was taken as the mean diameter of the colliding particles, with the *ω* parameter being the same as for Ag–Ag and He–Ag collisions. It should be noted that dimer–dimer collisions were not considered due to their very low probability.

For modeling Reactions (3)–(6) within the DSMC algorithm, the total collision energy (TCE) scheme was employed [36,53]. This scheme suggests that the equilibrium rate coefficients of the forward and reverse processes can be presented in the extended Arrhenius form as follows:

(7) $$K_{c}=\frac{K_{21,\mathrm{Ag}}}{K_{12,\mathrm{Ag}}}=\frac{K_{21,\mathrm{He}}}{K_{12,\mathrm{He}}}=AT^{B}\exp\left(-E_{a}/kT\right).$$

where $E_{a}$ is the activation energy, and *A* and *B* are constants. The parameters of Equation (7) can be evaluated using the equilibrium size distribution of silver clusters and dimer dissociation energy $E_{d}$ with *A* = 10.86 × 10^33^ m^−3^ K, *B* = −1, and $E_{a}=E_{d}$ = 1.67 eV [52,54]. The rate coefficients of forward processes (3) and (4) assuming the Arrhenius expression without an activation barrier can be expressed as follows [51]:

(8) $$K_{12,\mathrm{Ag}}=\alpha_{\mathrm{Ag}}T^{\beta_{\mathrm{Ag}}},\;K_{12,\mathrm{He}}=\alpha_{\mathrm{He}}T^{\beta_{\mathrm{He}}}$$

Reactions (3) and (4) can be considered as a two-step process [51]: (i) the formation of an excited quasi-dimer ${\mathrm{Ag}}_{2}^{*}$ in a paired Ag–Ag collision and (ii) the stabilization of the excited cluster by a collision with a third atom (helium or silver). Assuming that the lifetime *t_L_* of the quasi-dimer depends on the relative collision velocity as $t_{L}=H\sigma_{\mathrm{Ag}}^{1/2}/c_{\mathrm{Ag}}$ [51,55] (here, $\sigma_{\mathrm{Ag}}=\pi d_{\mathrm{Ag}}^{2}$ is the collision cross-section, $c_{\mathrm{Ag}}$ is the relative velocity of colliding Ag atoms, and *H* is a constant) and that any collision with a third atom leads to dimer stabilization, the rate constant of the direct reactions can be presented in the following form [55]:

(9) $$K_{12}=2^{\frac{11}{4}-\frac{3}{2}\omega_{\mathrm{Ag}}-\omega_{*}}\pi^{\frac{3}{2}}H\Gamma \left(\frac{5}{2}-\omega_{*}\right)\Gamma \left(\frac{9}{4}-\frac{3}{2}\omega_{\mathrm{Ag}}\right)d_{*}^{2}c_{*}^{2\omega_{*}-1}d_{\mathrm{Ag}}^{3}c_{\mathrm{Ag}}^{3\omega_{\mathrm{Ag}}-\frac{3}{2}}\mu_{2}^{\omega_{*}-1}\mu_{\mathrm{Ag}}^{\frac{3\omega_{\mathrm{Ag}}}{2}-\frac{3}{4}}k^{\frac{7}{4}-\omega_{*}-\frac{3}{2}\omega_{\mathrm{Ag}}}T^{\frac{7}{4}-\omega_{*}-\frac{3}{2}\omega_{\mathrm{Ag}}}$$

where $\omega_{*},\;d_{*},\;c_{*}$ are the reference viscosity index, diameter, and relative velocity, respectively, of the VHS model for a dimer–atom collision. $\mu_{2}=2m_{1}m_{\mathrm{Ag}}/\left(m_{1}+2m_{\mathrm{Ag}}\right)$ is the reduced mass of the atom–dimer system ($m_{\mathrm{Ag}}$ is the mass of a Ag atom, and $m_{1}$ is the mass of the third atom (Ag or He)), and $\mu_{\mathrm{Ag}}=m_{\mathrm{Ag}}/2$.

The parameter *H* was related to the lifetime of a quasi-dimer and could vary in fairly large limits. The minimal value of $H=H_{S}=1/\pi^{1/2}$ corresponded to a lifetime equal to the mean time between direct collisions of Ag monomers as hard spheres [51]. In this case, Equation (9) was reduced to $K_{12}=\xi d_{\mathrm{Ag}}^{5}{\left(kT/m_{\mathrm{Ag}}\right)}^{1/2}$ with a coefficient ξ of the order of 1 [56,57]. A value of $H>H_{S}$ assumed that the interaction between reacting particles was more complicated than that described with simple VHS mechanics and involved an orbiting motion of the particles with the formation of “long-lived” complexes [58,59,60] and, thus, a quasi-dimer had more time for stabilization. High *H* values appeared to be more appropriate for the considered conditions, as the experimental data on cluster formation could only be described with $H\gg H_{S}$ (see Section 4.1).

Based on Equation (9), we evaluated the following parameters in Equation (8) for the rate constants of forward reactions (3) and (4): *α*_Ag_ = 4.6 × 10^−46^*H* m^6^K^−0.5^s^−1^, *β*_Ag_ = 0.5, *α*_He_ = 2.6 × 10^−45^*H* m^6^ K^−0.42^ s^−1^, and *β*_He_ = 0.42. Two *H* values were mainly considered in the simulations: $H=H_{S}$ and *H* = 1000. The rate constants of reverse reactions (5) and (6) were obtained using the detailed balance principle from Equations (7) and (8).

The Larsen–Borgnakke model for the continuum energy spectrum [36] was used for energy exchange processes between dimers and atoms. The numbers of rotational and vibrational degrees of freedom for Ag_2_ were 2 and 1, respectively. The probability of the collisional exchange between the dimer translational and internal energies was adopted to be 0.1 [46,61].

## 4. Results and Discussion

### 4.1. Jet Expansion and Cluster Formation

**Figure 3:**
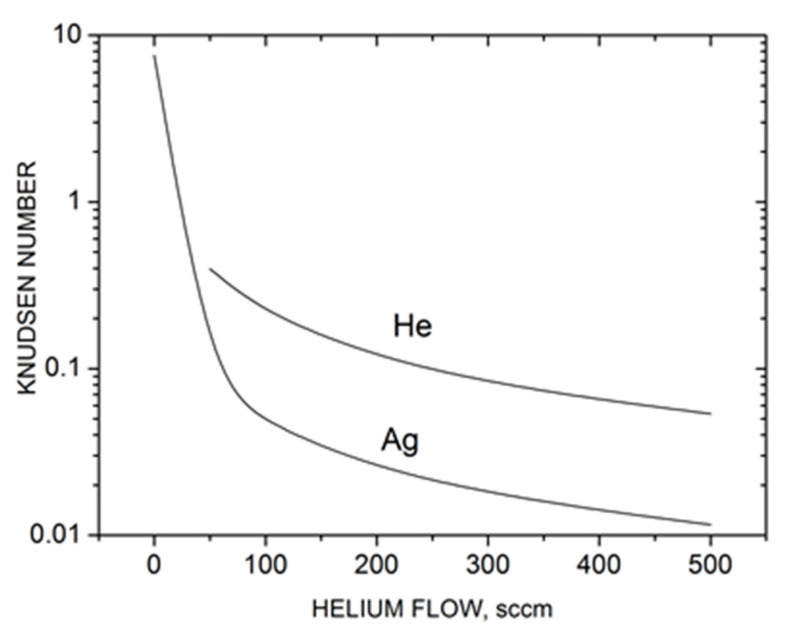
Characteristic Knudsen numbers Kn *= λ*_0_/*R*_0_ of silver and helium in the Ag-He jet based on the source parameters as a function of helium flow.

Both simulations and experiments were performed at a fixed source temperature of *T*_0_ = 1373 K with the carrier gas (helium) flow varied in the range of 0–500 sccm. To characterize the rarefaction degree of the Ag-He mixture under these conditions, it was convenient to consider two Knudsen numbers of Kn *= λ*_0_/*R*_0_, separately for Ag and He, with the mean free path *λ*_0_ determined from the source parameters. According to the VHS model, the mean free path of Ag atoms was as follows [36]:

(10) $$\lambda_{0,\mathrm{Ag}}={\left[\sqrt{2}\pi d_{\mathrm{Ag}}^{2}n_{0,\mathrm{Ag}}{\left(\frac{T_{ref}}{T_{0}}\right)}^{\omega_{\mathrm{Ag}}-\frac{1}{2}}+\pi d_{\mathrm{Ag}-\mathrm{He}}^{2}n_{0,\mathrm{He}}{\left(\frac{T_{ref}}{T_{0}}\right)}^{\omega_{\mathrm{Ag}-\mathrm{He}}-\frac{1}{2}}{\left(1+\frac{m_{\mathrm{Ag}}}{m_{\mathrm{He}}}\right)}^{1/2}\right]}^{-1}$$

where $n_{0,\mathrm{He}}$ is the He density in the central cylindrical part of the source. The $\lambda_{0}$ value for helium atoms could be determined in a similar way. The calculated Knudsen numbers are shown in Figure 3 as a function of He flow. Without the carrier gas ($Q_{\mathrm{He}}$ = 0), the silver vapor expansion occurred in a nearly free molecular regime (Kn_Ag_ > 1), and thus, collisional clustering in the jet was unlikely. However, with the introduction of helium in the source, the Kn_Ag_ value dropped quickly due to frequent Ag–He collisions and a relatively low Ag mean thermal velocity (factor ${\left(1+m_{\mathrm{Ag}}/m_{\mathrm{He}}\right)}^{1/2}$ in the second term of Equation (10)). As a result, throughout the studied helium flow rates, the silver expansion regime could be characterized as transient between free molecular and hydrodynamic (continuous) regimes, where the latter could be considered at Kn < ~10^−3^ [62]. This is important for the present study, as the probability of silver gas-phase cluster formation increases strongly in the He-Ag mixture jet. Helium expansion also occurred in the transient regime, although at slightly lower Knudsen numbers (Figure 3). We could also formally introduce a total Knudsen number Kn_mix_ for the entire Ag-He mixture determined from the density-averaged mean free path. Since the helium density in the source was considerably higher than that of silver, Kn_mix_ ≈ Kn_He_.

**Figure 4:**
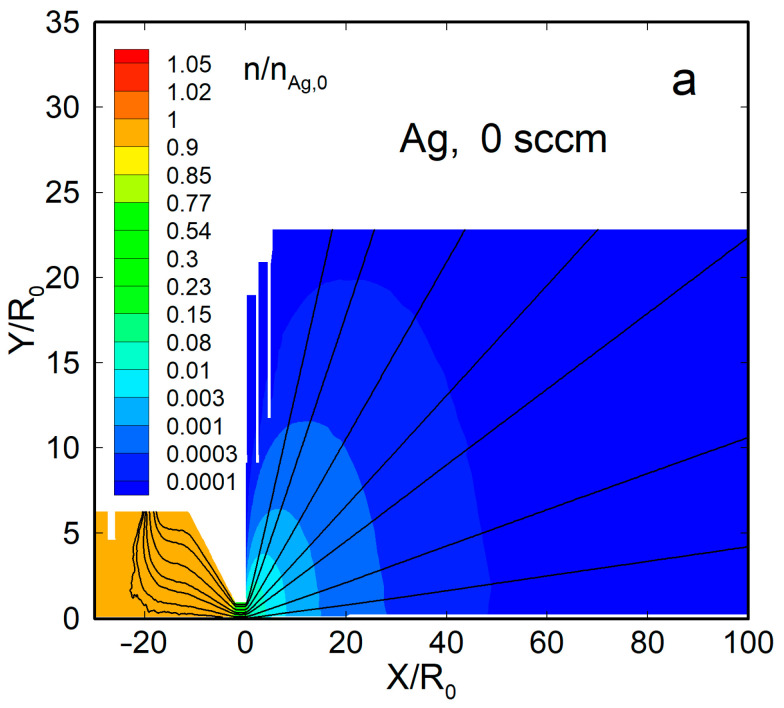

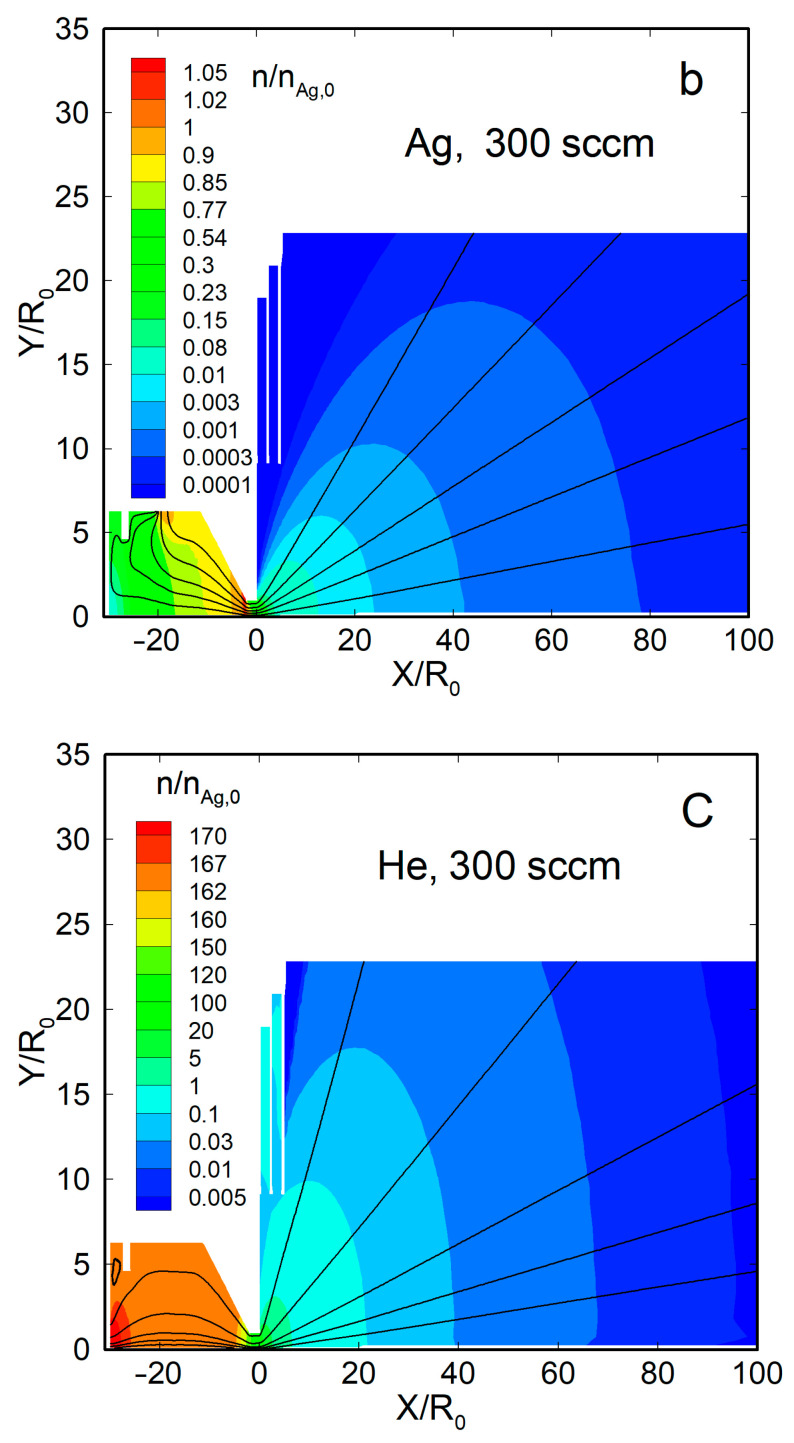
The density fields and streamlines of Ag and He atoms in the jets: (**a**) Ag ($Q_{\mathrm{He}}$ = 0), (**b**) Ag ($Q_{\mathrm{He}}$ = 300 sccm), and (**c**) He ($Q_{\mathrm{He}}$ = 300 sccm). The density values were normalized to that of silver vapor in the source, at $n_{0,\;\mathrm{Ag}}$ = 2.1 × 10^14^ cm^−3^.

Figure 4 shows the density flow fields and streamlines for two representative cases, a pure silver jet and an Ag-He jet, at $Q_{\mathrm{He}}$ = 300 sccm. When no helium was introduced, the Ag vapor was uniformly distributed within the source, with a significant gradient only near the nozzle exit (Figure 4a). Beyond the nozzle, the vapor expanded nearly spherically, and the density dropped by four orders of magnitude at a distance from the nozzle exit of *X*/*R*_0_ ≈ 50. The thermal shields did not disturb the main flow and affected only the jet periphery.

With the carrier gas, the Ag density flow field was changed significantly, both inside the source and in the jet (Figure 4b). The density was reduced near the helium inlet (as the Ag vapor was forced out by the gas) and, as a result, was maximized inside the source near the nozzle, where it even exceeded the saturation $n_{0}$ value [63]. Beyond the nozzle, in contrast to the nearly spherical free molecular expansion, the silver jet was now rather forward-directed, and its density decreased slower with distance than in the pure Ag jet. The helium density field flow was different from that of Ag (Figure 4c). It was uniform inside the VGJD source, while the He jet expansion was directed, rather, to the periphery, and the density on the jet axis dropped with distance quicker than that of Ag. As a result, the jet axis was enriched with heavier species of the Ag-He mixture during expansion, as demonstrated in recent simulations [63].

**Figure 5:**
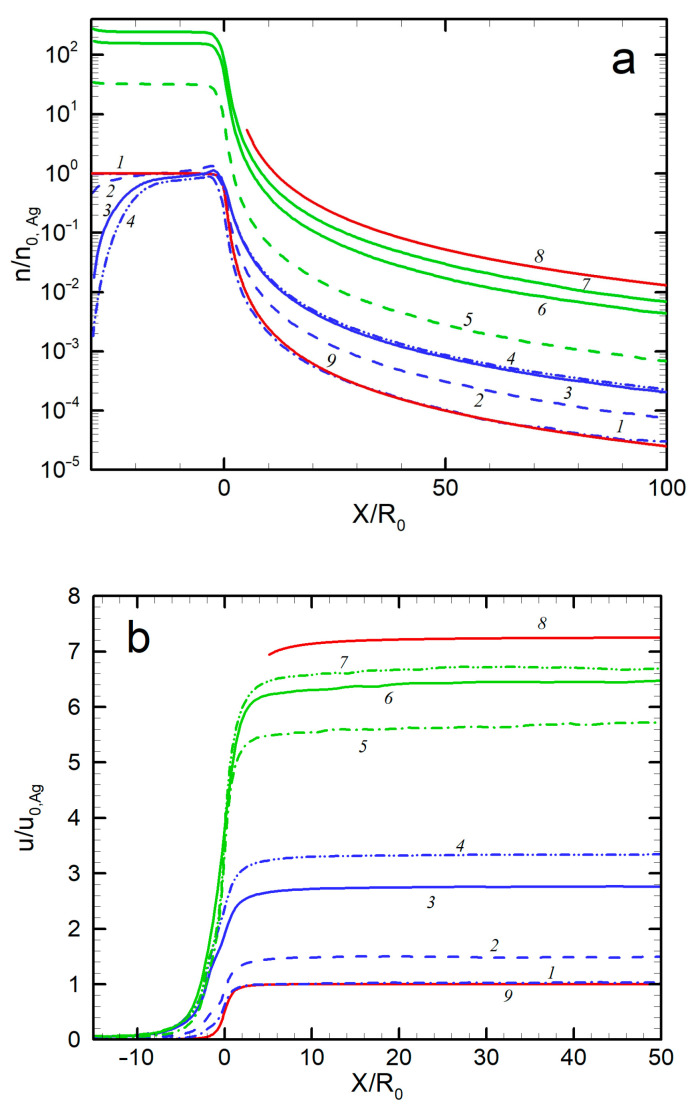

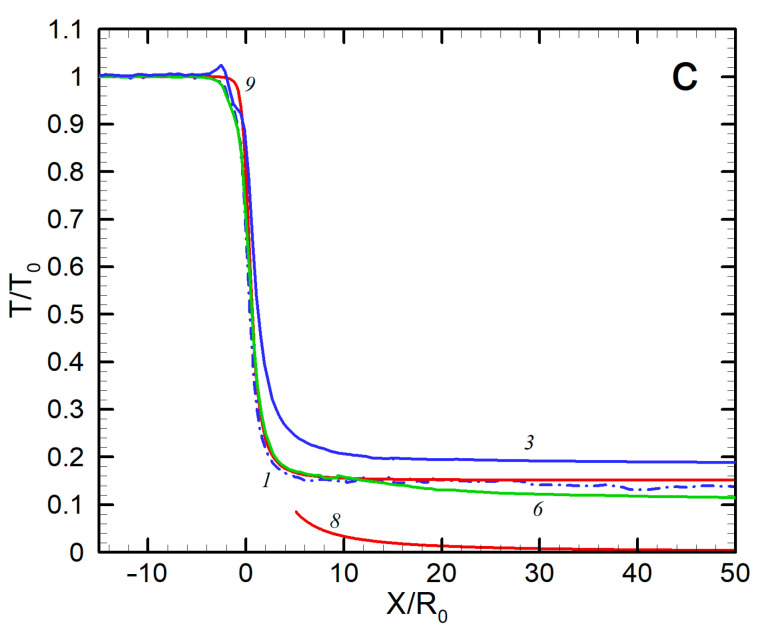
Axial distributions of density (**a**), velocity (**b**), and temperature (**c**) for Ag and He: 1—Ag, no He; 2—Ag, 50 sccm; 3—Ag, 300 sccm; 4—Ag, 500 sccm; 5—He, 50 sccm; 6—He, 300 sccm; 7—He, 500 sccm; 8—He, no Ag (hydrodynamic expansion); 9—Ag, no He (free molecular regime).

The axial distributions of the jet gas dynamic parameters (density, velocity, and temperature) of both species of the Ag-He mixture are shown in Figure 5 for different expansion conditions. For comparison, the parameters for two extreme cases, a free molecular silver flow and a continuous helium jet in the hydrodynamic regime (Kn << 1), are shown. The first regime was described with well-established solutions [62,64], while for the latter case, the solution [65] for a dense, inviscid wind tunnel jet was used. Several results can be remarked on here. First, the parameters of the pure Ag jet precisely followed the free molecular solution. In particular, the terminal velocity $u_{0,\mathrm{Ag}}$ and temperature $T_{\mathrm{Ag}}$ were close to the values for collisionless expansion [62]: $u_{0,\mathrm{Ag}}={\left(8kT_{0}/\pi m_{\mathrm{Ag}}\right)}^{1/2}$ and $T_{\mathrm{Ag}}=\left(1-8/3\pi \right)T_{0}$. Second, the introduction of the carrier gas accelerated Ag atoms in the jet, and the higher the He flow, the stronger the acceleration (Figure 5b). However, Ag atoms were still slower than helium atoms, and even in the densest regime considered here ($Q_{\mathrm{He}}$ = 500 sccm), there was considerable velocity slip between Ag and He, a well-known effect for rarefied adiabatic expansion of gaseous mixtures [66,67,68]. The relative terminal velocity slip $\left(u_{\mathrm{He}}-u_{\mathrm{Ag}}\right)/u_{\mathrm{He}}$ was ~0.74 for $Q_{\mathrm{He}}$ = 50 sccm (Kn_mix_ = 0.39) and ~0.5 for $Q_{\mathrm{He}}$ = 300 sccm (Kn_mix_ = 0.053). It was previously demonstrated that the velocity slip was nearly proportional to the Knudsen number for relatively small differences in molecular masses (Ne-He and Ar-He systems [68]), but the Knudsen dependence was much weaker for disparate mass mixtures (e.g., for a Xe-He jet [67]). The latter case was obviously realized in our Ag-He jet, where the relative velocity slip decreased by only a factor of ~1.5 when the Knudsen number dropped by approximately an order of magnitude. Therewith, the terminal He velocity nearly reached the maximal hydrodynamic value for continuous inviscid expansion [65] of $u_{\max}={\left(2\gamma kT_{0}/\left(\gamma -1\right)m_{\mathrm{av}}\right)}^{1/2}$, where γ = 5/3 is the specific heat ratio and $m_{\mathrm{av}}=(m_{\mathrm{He}}n_{0,\mathrm{He}}+m_{\mathrm{Ag}}n_{0,\mathrm{Ag}})/\left(n_{0,\mathrm{He}}+n_{0,\mathrm{Ag}}\right)$ is the average atomic mass of the mixture in the source. Thus, for $Q_{\mathrm{He}}$ = 500 sccm, $m_{\mathrm{av}}$ = 4.4 u and $u_{\max}$ = 6.9$u_{0,\mathrm{Ag}}$, while the calculated $u_{\mathrm{He}}$ = 6.7$u_{0,\mathrm{Ag}}$ (Figure 5b). Such a small deviation from the inviscid behavior for our transition regimes was not unexpected since the main transfer from thermal to kinetic energy occurred at early expansion stages where the collision frequency was still sufficient for the transfer to reach hypersonic velocities, as demonstrated with nonequilibrium helium jets [69].

The third emerging result was the translational temperature behavior in the jet (Figure 5c). The temperature was determined as a measure of the spread of atom thermal velocity relative to the mean flow velocity. We can see that, for all the considered conditions, the temperature dropped quickly at early expansion stages and then froze out at relatively short distances from the nozzle (at *X*/*R*_0_ = 10–20 for silver and 30–40 for He, depending on the helium flow rate) at values far higher than those given by the continuous inviscid solution [60]. This agreed well with the temperature measurements in helium-free jets, where deviation from the equilibrium was observed at similar distances from the nozzle even at lower Knudsen numbers. To correlate the equilibrium breakdown onset with the jet parameters, Bird introduced an empirical rarefaction parameter based on a logarithmic time derivation of the density [36]:

(11) $$P_{B}=\frac{1}{\nu }\frac{d(\ln n)}{dt}$$

where *ν* is the collision frequency. It has been found [36,69] that the onset of the equilibrium breakdown occurs at *P_B_* ≈ 0.1, while at *P_B_* ≈ 1 the jet temperature is almost frozen out. The parameter *P_B_* is, in fact, a local Knudsen number in the flow and, assuming spherical-source-like behavior of the density near the jet axis, it can be shown that the parameter is related to the source Knudsen number Kn_0_ (Figure 3) as *P_B_* = Kn_0_(*X*/2*R*_0_). Then, we can evaluate the distance with *P_B_* = 1, where the freezing process terminated. For instance, for the jet with $Q_{\mathrm{He}}$ = 300 sccm, this occurs at *X*/*R*_0_ ≈ 25, which is in good agreement with the simulated temperature distribution (Figure 5c). The temperature-freezing process is responsible for the obtained velocity slip (Figure 5b) since otherwise the frozen thermal vapor energy would appear as atom kinetic energy, resulting in higher Ag velocities. It is also notable that the temperature-freezing data allowed us to estimate the upper limits of the jet region where the clustering process can potentially occur since, beyond the point of *P_B_* ≈ 1, the expansion is collisionless and neither cluster formation nor growth is possible.

**Figure 6:**
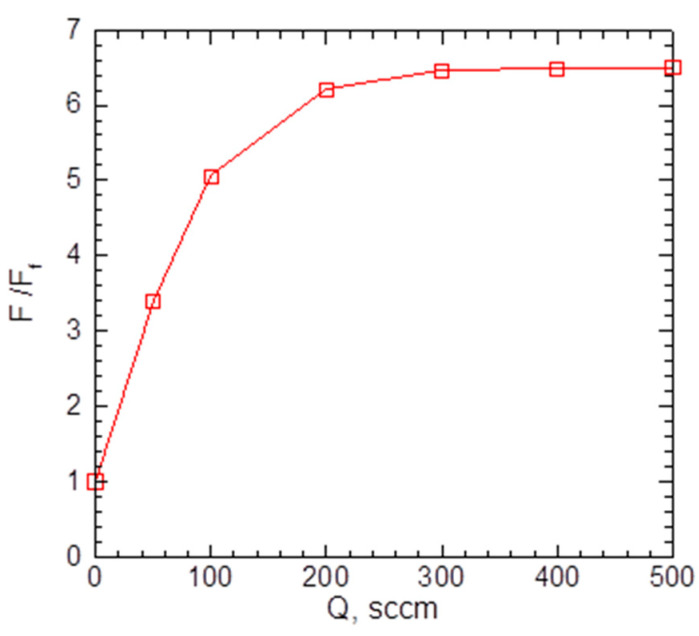
Calculated Ag flux through the nozzle as a function of He flow. The values were normalized to the atom flux for a pure Ag jet at $F_{f}$ = 1.35 × 10^18^ cm^−2^s^−1^.

As the introduction of the carrier gas into the VGDJ source increased both silver density and velocity near the nozzle exit (Figure 4 and Figure 5), the resulting Ag flux through the nozzle was much higher compared to the pure metal jet. Figure 6 shows the flux as a function of helium flow. As seen, with helium, the Ag flux could be increased by a factor of ~7 at the same source temperature. This enabled a strong increase in the film deposition rate with the VGJD method, as demonstrated in Section 4.2. The simulations show, however, that the Ag flux reached a saturation value at a certain He flow (approximately 300 sccm under the considered conditions) and did not increase with a further rise in the $Q_{\mathrm{He}}$. The saturation was due to the backscattering of Ag atoms in collisions with He atoms, resulting in Ag recondensation on the metal surface [29], an effect which was stronger at higher $Q_{\mathrm{He}}$ values. It should be noted that the maximal obtained Ag flux corresponded to a silver mass flux of about 0.1 mg/s, which was still at least two orders of magnitude lower than typical mass fluxes in the nozzle CBD method [24], i.e., VGJD was a considerably more material-saving technique.

The possibility of cluster formation in metal vapor nozzle expansions can be estimated based on Hagena’s scaling law [24,28,70]. For pure metal jets from an orifice (i.e., sonic nozzle), the law predicts that clustering is correlated by the reduced scaling parameter $\Gamma_{*}$, which combines the source conditions and material properties:

(12) $$\Gamma_{*}=\Gamma /\Gamma_{\mathrm{ch}}=n_{0}D_{0}^{0.85}T_{0}^{-1.29}/\Gamma_{\mathrm{ch}}$$

where $D_{0}=2R_{0}$, and $\Gamma_{\mathrm{ch}}$ is a material-dependent constant that is tabulated for silver vapor as $\Gamma_{\mathrm{ch}}$ = 6.0 × 10^14^ m^−2.15^K^−1.29^ [28]. According to [24,28], in any gas/vapor jet, the clustering onset occurs at $\Gamma_{*}\approx$ 200, while for conditions with $\Gamma_{*}$ < 200, no cluster formation is predicted. In the considered regime of the pure silver jet (*T*_0_ = 1373 K, $n_{0}$ = 2.1 × 10^14^ cm^−3^ [44], and $D_{0}$ = 3 mm), the parameter $\Gamma_{*}\approx$ 0.3, i.e., it is much smaller than the critical value of 200, and thus, the jet is too rarefied for clustering to start.

The parameter $\Gamma_{*}$ and criterion $\Gamma_{*}$ > 200 are not directly applicable to the Ag-He mixture jet considered here. However, we can argue that, if a light carrier gas is used, several factors would strongly promote the condensation process in the vapor jet. First, the frequency of triple-clustering collisions (see, e.g., Equation (4)) increases where helium atoms serve as a third body, allowing the removal of the heat of condensation and overcoming the bottleneck of the nucleation process, the formation of dimers [31]. For instance, in experiments with gas mixture jets, diluting argon with helium down to a 5% mole fraction allowed the reduction of the Ar partial pressure in the source by a factor of ~4 to obtain the same size of Ar*_n_* clusters compared with a pure Ar jet [31]. In our case of an Ag-He mixture with a larger ratio of atomic masses and a lower silver mole fraction (Figure 5a), the effect can be even stronger. Second, a narrower expansion angle of the silver jet in the presence of a helium carrier gas (cf. Figure 4a,b) hels to maintain a higher Ag density in the near-axis region, which is favorable for clustering. Such a forward-directed expansion is similar to a flow in a conical supersonic nozzle, where an equivalent nozzle throat diameter in Equation (12) is proportional to $D_{0}/\tan\alpha$ (where α is the semiangle of the cone) and, thus, cluster formation is more efficient [24,31]. Third, the much higher total pressure in the source through introducing the carrier gas is essential to reduce the nozzle boundary layer, which is also beneficial for metal clustering [18]. It is difficult to evaluate the combined effect of all the above factors for the considered conditions, but the formation of small silver clusters at sufficiently high helium flows seems likely to occur.

**Figure 7:**
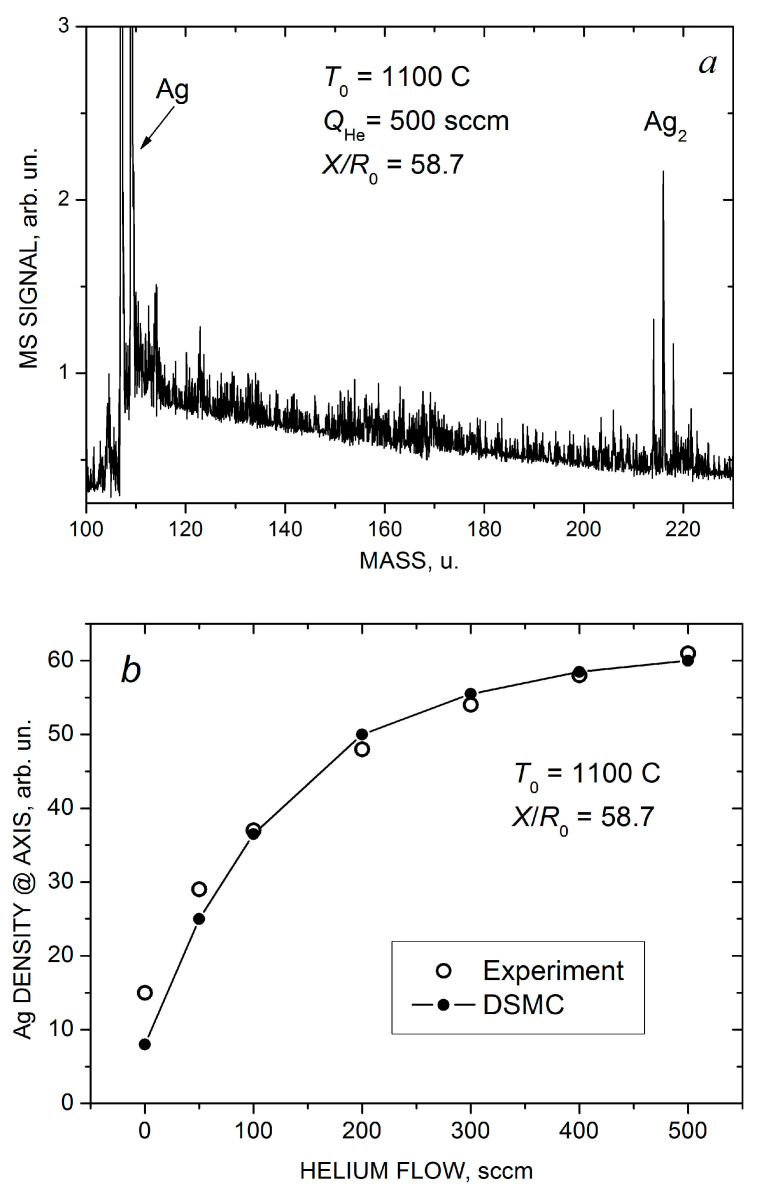

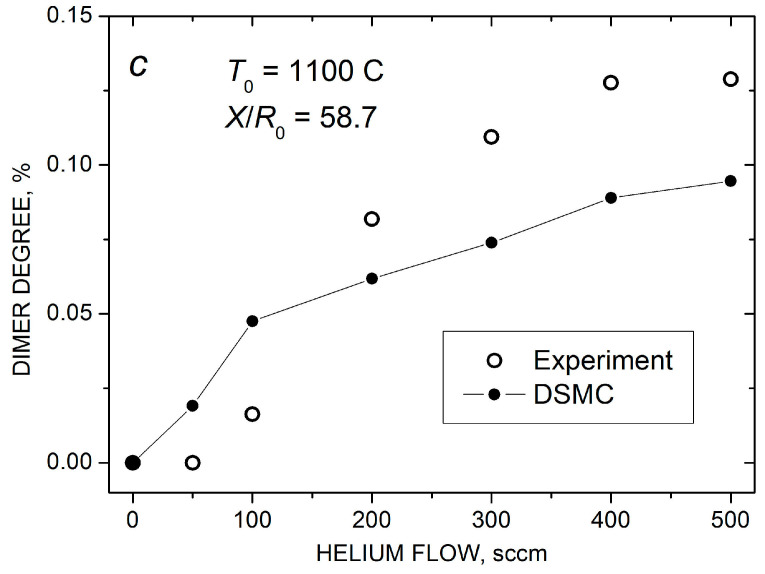
Mass spectra of silver species in the Ag-He jet at $Q_{\mathrm{He}}$ = 500 scm and *X*/*R*_0_ = 58.7 (**a**). Measured (open symbols) and calculated (solid-line-connected symbols) Ag atom density (**b**) and Ag_2_ dimer fraction in the jet axis (**c**) as a function of He flow.

Indeed, based on the mass spectrometric measurements we found that the pure silver jet and jets with low helium flow ($Q_{\mathrm{He}}$ < 100 sccm) consisted exclusively of Ag atoms, and no silver clusters were detected. However, at higher helium flows, Ag_2_ dimers were registered with confidence in the jet, and their concentration increased with $Q_{\mathrm{He}}$. A typical mass spectrum of silver species obtained at $Q_{\mathrm{He}}$ = 500 sccm is illustrated in Figure 7a (silver has two isotopes, ^107^Ag 51.8% and ^109^Ag 48.2%, and the dimer signal represents a characteristic tree-peak structure). Note that the jet was sampled for the MS analysis quite far from the nozzle, at *X/R*_0_ = 57.8, where the flow was collisionless and, thus, the final jet composition was measured. At the highest used helium flows, traces of Ag_3_ trimers were also detected [22] (not shown in Figure 7a).

Figure 7b,c show the measured and calculated on-axis density $n_{\mathrm{Ag}}$ of Ag atoms and the final mole fraction $x_{2}=n_{2}/\left(n_{\mathrm{Ag}}+n_{2}\right)\;\approx n_{2}/n_{\mathrm{Ag}}$ of Ag_2_ dimers in the jet as a function of helium flow. Here, $n_{2}$ is the dimer density, and we assumed that our mass spectrometer with an electron gun ionizer was a density-sensitive detector [43,71]. In estimations of the experimental values of the dimer fraction, the almost twofold difference in the electron impact ionization cross-sections of silver atoms and dimers [72] was taken into account. As demonstrated above (Figure 4a,b and Figure 5a), the introduction of helium in the VGJD source resulted in the enhancement of the Ag density in the near-axis region, but the dependence was nearly saturated at fairly high He flows ($Q_{\mathrm{He}}$ > 300 sccm). The simulated $n_{2}\left(Q_{\mathrm{He}}\right)$ dependence agreed nicely with the MS measurements (Figure 7b).

The increase in the dimer concentration at $Q_{\mathrm{He}}$ > 100 sccm was even stronger than that for $n_{\mathrm{Ag}}$, and at $Q_{\mathrm{He}}$ > 300 sccm, the dimer abundance exceeded 0.1% of that for atoms (Figure 7c), which could be considered as a high value for our rarefied flows. However, again, with a further increase in $Q_{\mathrm{He}}$, the final Ag_2_ fraction was almost saturated (at least according to the MS data), even though the frequency of the stabilizing Ag_2_–He collisions continued to increase. This correlated with the saturations of the on-axis Ag atom density and the Ag flux through the nozzle (Figure 6) and indicated that the Ag–Ag pair collisions were, rather, a limiting factor for silver dimer formation under these conditions. The DSMC simulations predicted the same level of the dimer fraction and were in reasonable agreement with the measurements, although the calculated $x_{2}\left(Q_{\mathrm{He}}\right)$ dependence was somewhat weaker than the experimental one (Figure 7c). It should be noted that, in the simulations, dimers were only observed in the Ag-He mixtures with sufficiently large values of the *H* parameter (*H* > 10, see Equation (9)). The data shown in Figure 7c were obtained with *H* = 1000, which provided the best agreement with the experiment. This suggested that a metastable dimer (or quasi-dimer) has a fairly long lifetime of the order of 10^−9^–10^−10^ s during the orbiting motion of the colliding particles [58,59,60].

**Figure 8:**
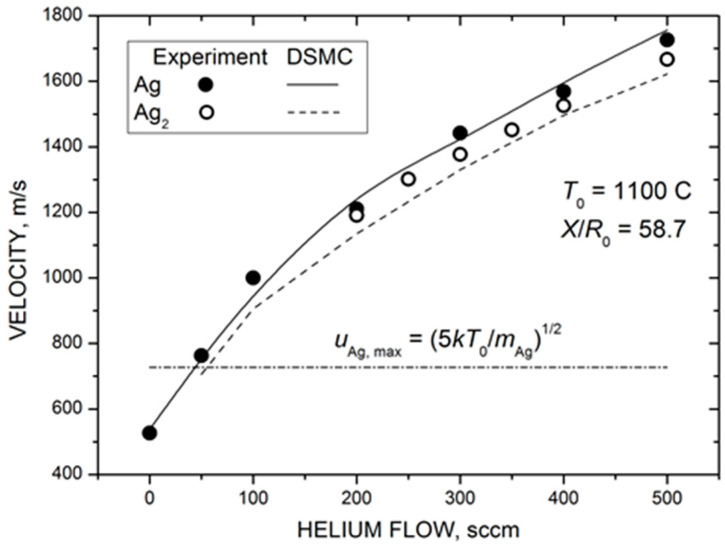
Measured (symbols) and calculated (lines) mean velocities of silver atoms and dimers at the jet axis at a distance of 88 mm from the nozzle exit as a function of helium flow. The dot-dashed line shows the maximal jet velocity in the supersonic expansion of pure Ag vapor in the hydrodynamic regime of $u_{\mathrm{Ag},\;\max}={\left(2\gamma kT_{0}/\left(\gamma -1\right)m_{\mathrm{Ag}}\right)}^{1/2}$ = 727 m/s.

Figure 8 shows the measured and calculated mean velocities of silver atoms and dimers in the Ag-He jet as a function of helium flow. Both silver species were strongly accelerated by the carrier gas, and their terminal velocities in the jet were considerably higher than the maximal flow velocity attainable in the adiabatic expansion of pure silver vapor $u_{\mathrm{Ag},\max}={\left(2\gamma kT_{0}/\left(\gamma -1\right)m_{\mathrm{Ag}}\right)}^{1/2}$ when the vapor thermal energy was completely converted into the kinetic energy (dot-dashed line in Figure 8). In contrast to the Ag density, the velocity values did not saturate at high $Q_{\mathrm{He}}$ values but increased almost linearly in the studied He flow range. This was not surprising since, even at the highest load of the carrier gas here ($Q_{\mathrm{He}}$ = 500 sccm), the velocity slip between Ag and He atoms was still significant (Figure 5b) and, thus, there was room for silver particle acceleration with further increase in the frequency of Ag–He collisions. The agreement between the measured and calculated velocity values was excellent, considering that no adjustable parameters were used for simulations in this case.

The most remarkable result of Figure 8 was the almost identical velocities of silver atoms and dimers throughout the studied $Q_{\mathrm{He}}$ range. This indicates that the observed Ag_2_ dimers were likely formed due to the condensation process in the expanding Ag-He jet and were not directly ejected from the nozzle walls, as previously suggested for metal clusters [73,74]. In the latter case, a considerable velocity slip between Ag and Ag_2_ would be expected for our rarefied jets due to the large difference in masses of these species [68]. In contrast, clusters produced in the gas-phase aggregation reactions in the expanding flows typically have the same (or very close) velocities for wide ranges of cluster sizes [75,76,77]. We cannot totally exclude a contribution of the direct emission to the dimer MS signal in our experiments, but the gas-phase aggregation in the jet (Equations (3) and (4)) appeared to be the main formation mechanism.

**Figure 9:**
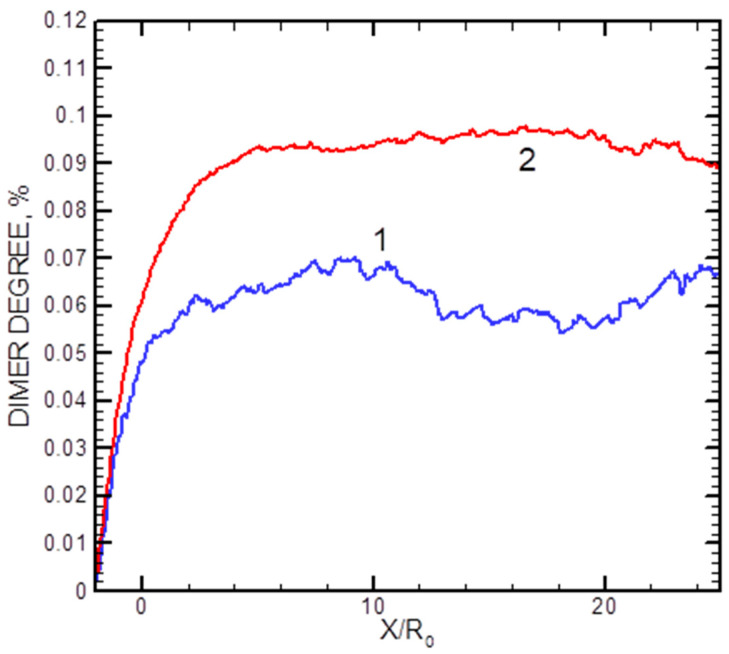
Axial distributions of the Ag_2_ dimer mole for helium flows of 200 sccm (1) and 500 sccm (2).

The calculated axial distributions of the dimer fractions *x*_2_ for two helium flows are shown in Figure 9. In the densest studied jet with $Q_{\mathrm{He}}$ = 500 sccm, the dimer fraction saturated at *X*/*R*_0_ ≈ 7, while in a more rarefied regime with $Q_{\mathrm{He}}$ = 200 sccm, it reached its final value at *X*/*R*_0_ ≈ 3 (although the statistics were rather poor in the latter case). The data illustrate that the dimer formation process occurred in the jet at early expansion stages, and then the cluster fraction was frozen out.

### 4.2. Deposition of Jet Particles

**Figure 10:**
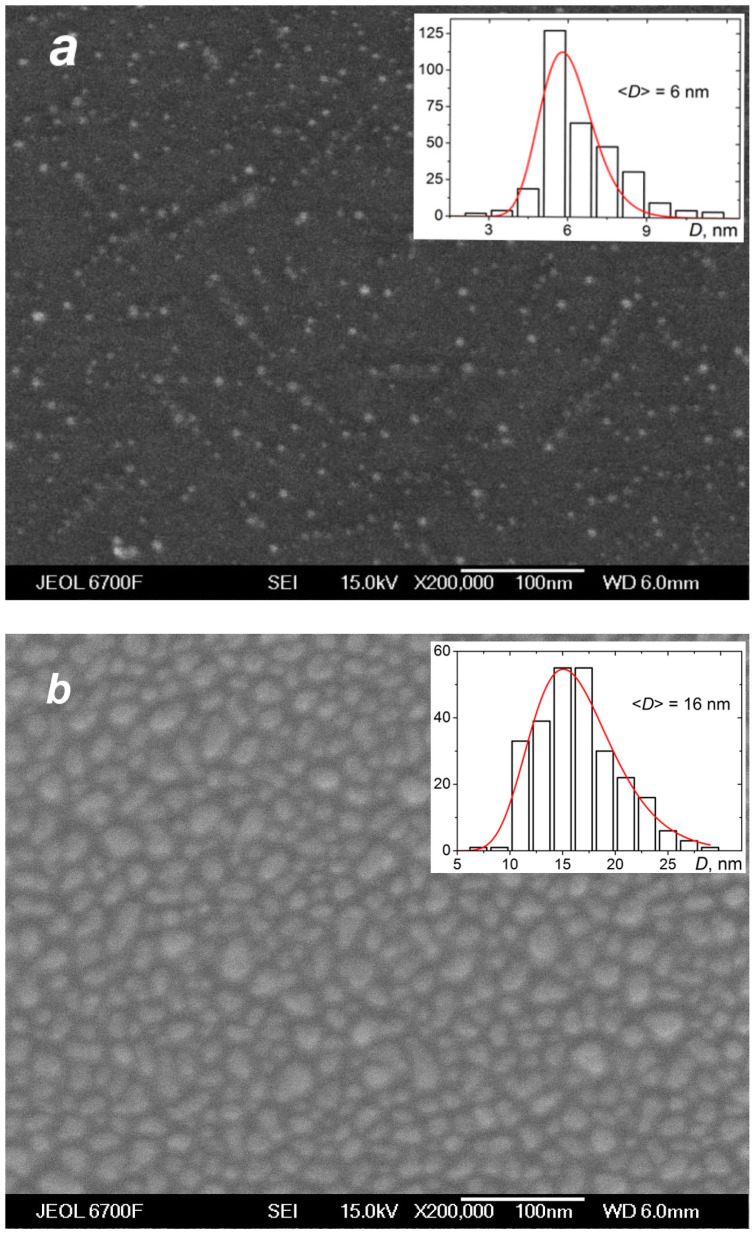
SEM images of silver films deposited on silicon substrates in atomic (**a**) and cluster (**b**) VGJD regimes with helium flows of 0 and 200 sccm, respectively, for identical deposition times of 15 s. The source temperature was 1373 K, and the substrates were located 310 mm from the nozzle exit (*X*/*R*_0_ = 207). The mass thicknesses of the films were below 1 nm for (**a**) and 12 nm for (**b**). The insets show the nanoparticle size distributions with the mean particle diameter <*D*> indicated. The solid lines correspond to fits of the distributions with a log-normal function.

The deposition of the species from the silver jet was performed under the same expansion conditions as in the studies of the jet (Section 4.1) to correlate characteristics of the produced films with the flow parameters and, in particular, to reveal the role of the small jet clusters in the film formation process. Figure 10 shows typical SEM images of films obtained with a purely atomic jet without clusters ($Q_{\mathrm{He}}$ = 0, Figure 10a) and in the cluster regime ($Q_{\mathrm{He}}$ = 200 sccm, Figure 10b) for the same deposition time of 15 s. In the first case, most of the substrate surface remained uncovered by silver, with Ag atoms distributed in small groups of islands that were formed on the surface due to atom diffusion and aggregation when island nucleation centers were provided by thermal fluctuations and defects [22,78,79]. The average island size was ~6 nm (the size distribution is shown in the inset of Figure 10a). The deposition rate with the pure Ag jet was low, and the mass thickness of the produced film was below the detection limit with the optical method, which was about 1 nm [45]. When the helium carrier gas was introduced, both the density and velocity of Ag atoms at the jet axis were increased (Figure 7b and Figure 8), resulting in a strong enhancement of the deposition rate. A film produced with $Q_{\mathrm{He}}$ = 200 sccm during the same deposition time was an order of magnitude thicker than that obtained without helium (Figure 10b). The film was still nanostructured with an island structure, but the nanoparticles were much denser, and their mean size was almost threefold higher (~16 nm, see inset in Figure 10b).

**Figure 11:**
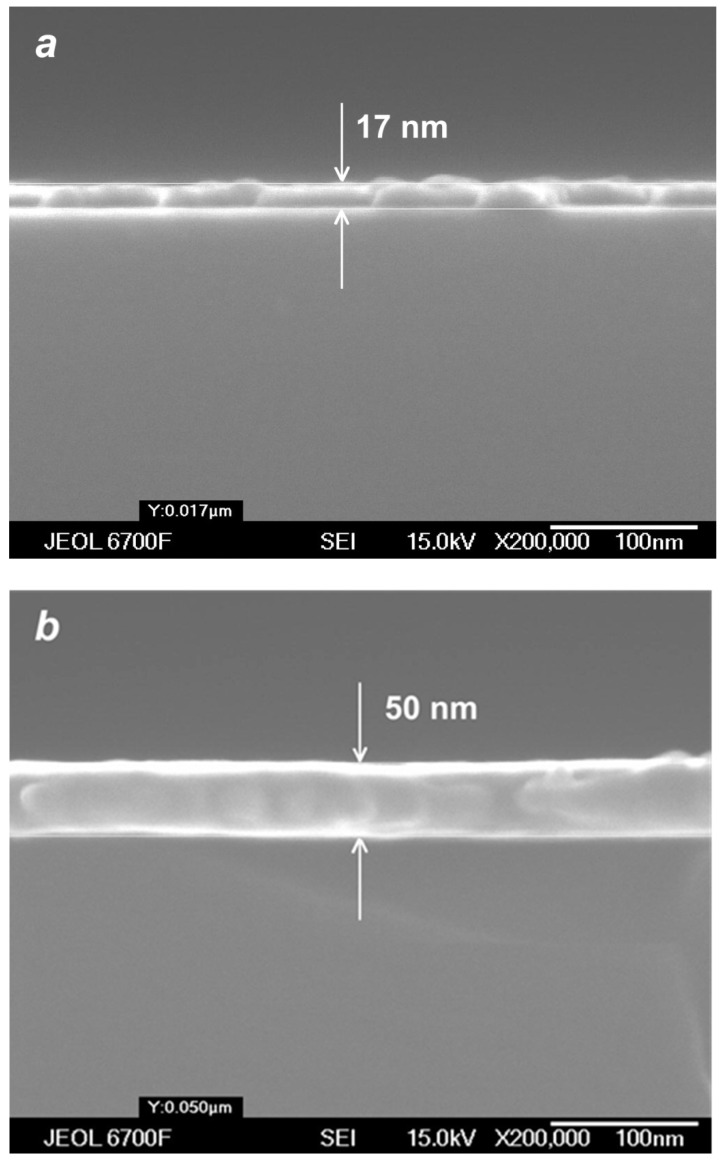
SEM images of cross-sections of silver films obtained in cluster VGJD regimes with $Q_{\mathrm{He}}$ = 100 sccm (**a**) and 500 sccm (**b**) for the same deposition time of 40 s.

**Figure 12:**
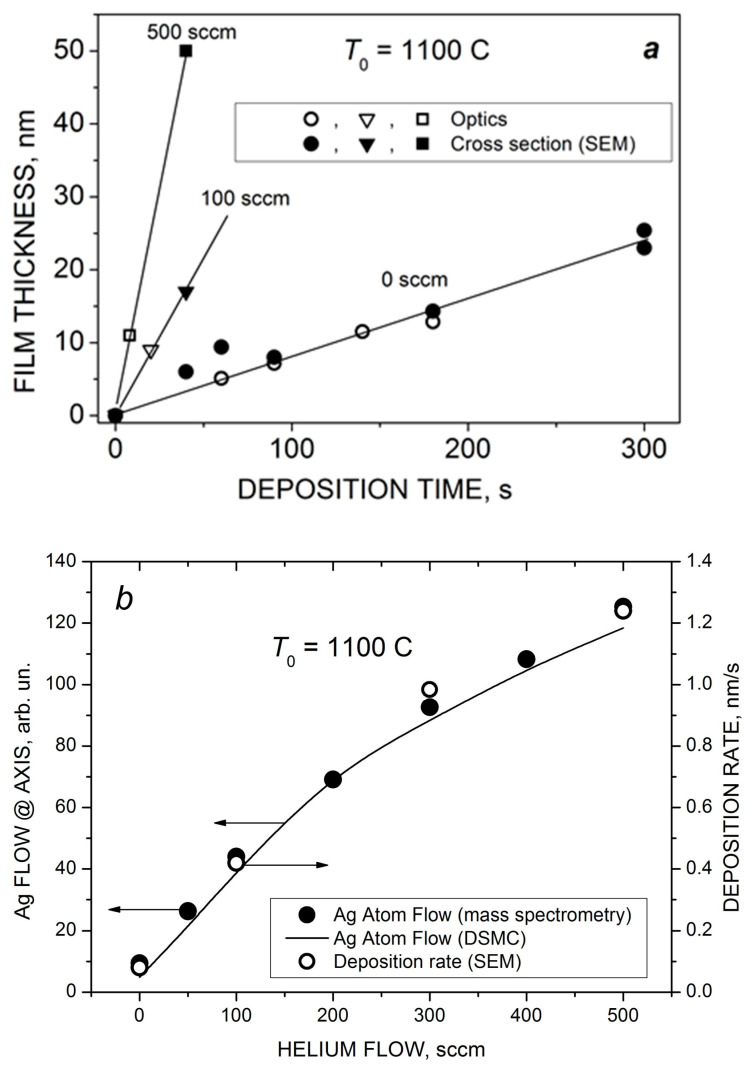
(**a**) The thickness of VGJD-produced Ag films at different helium flows as a function of deposition time. The thicknesses were measured using the optical method (quartz substrate) and SEM (silicon substrate). (**b**) Comparison of the $Q_{\mathrm{He}}$ dependencies of the Ag atom flow $n_{\mathrm{Ag}}u_{\mathrm{Ag}}$ at the jet axis (measured and calculated 88 mm from the nozzle) and the film deposition rate (for Si substrates located 310 mm from the nozzle).

With a further increase in the deposition time and/or helium flow, continuous Ag films could be obtained using the VGJD method. Figure 11 shows cross-sectional SEM images of such films produced in cluster regimes of silver jet expansion with low and high helium flows. The obtained continuous film was used for calibration of the optical transmittance method [45] to determine the mass-equivalent film thickness of the nanostructured films. The thicknesses of silver films produced with different VGJD regimes and measured using both techniques are shown in Figure 12a. The data directly demonstrate that an increase in the carrier gas flow led to a dramatic enhancement of the film deposition rate. A practical result of this enhancement was the possibility to perform the deposition of sufficiently thick films at fairly large distances from the nozzle for reasonably short times and, thus, to obtain highly uniform films over large areas, as was recently demonstrated with VGJD-produced substrates for surface-enhanced Raman spectroscopy (SERS) [22]. It should be noted that, at a fixed source temperature, the film thickness could be controlled independently by two parameters: the flow of the carrier gas and the deposition time. The VGJD deposition rates expressed in deposited atomic monolayers (MLs) per second corresponded, depending on the regime, to values in the range of 0.3–3 ML/s (assuming that the silver atomic layer was ~0.3 nm [43]). Therefore, the VGJD method exceeded in productivity by at least two orders of magnitude such conventional deposition techniques as molecular beam epitaxy (MBE) and pulsed-laser deposition (PLD) with their typical mean deposition rates of ~10^−3^ and 10^−2^ ML/s, respectively [33]. It should be noted that VGJD-produced films were completely free of droplets and microparticles (Figure 10 and Figure 11), the ejection of which represents a serious drawback of the PLD technique [4,16].

The obtained deposition rate as a function of helium flow is shown in Figure 12b in comparison with the measured (mass spectrometry) and calculated (DSMC) flows *F*_Ag_ of silver atoms at the jet axis. The *F*_Ag_ value was determined as a product of the density and velocity of silver atoms obtained at *X*/*R*_0_ = 58.7 (Figure 7b and Figure 8). In contrast to the Ag flow at the nozzle exit, which was saturated at around 200 sccm (Figure 6), the particle flow in the jet continued to grow with further increase in the helium flow due to two processes occurring during the Ag-He mixture’s supersonic expansion that were favorable for deposition: enrichment of the near-jet region with Ag atoms and their acceleration by helium. The mass spectrometric and DSMC data were in excellent agreement with the deposition rate, even though the latter was obtained at a larger distance from the nozzle. This is because all the processes in the jet were frozen out beyond *X*/*R*_0_ = 58.7, and the background pressure was low enough to avoid considerable scattering of the Ag atoms by ambient molecules and, thus, to reduce the atom flow, as took place in [29].

**Figure 13:**
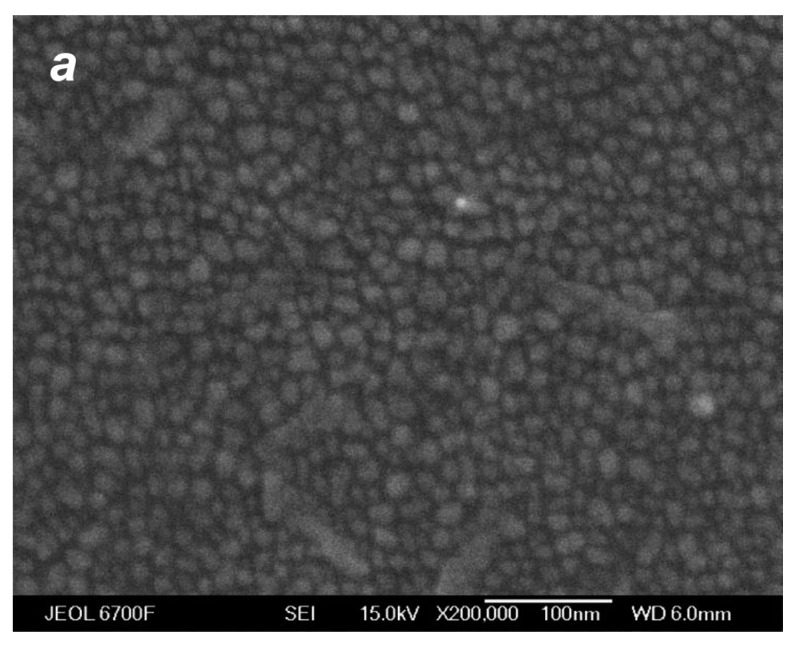

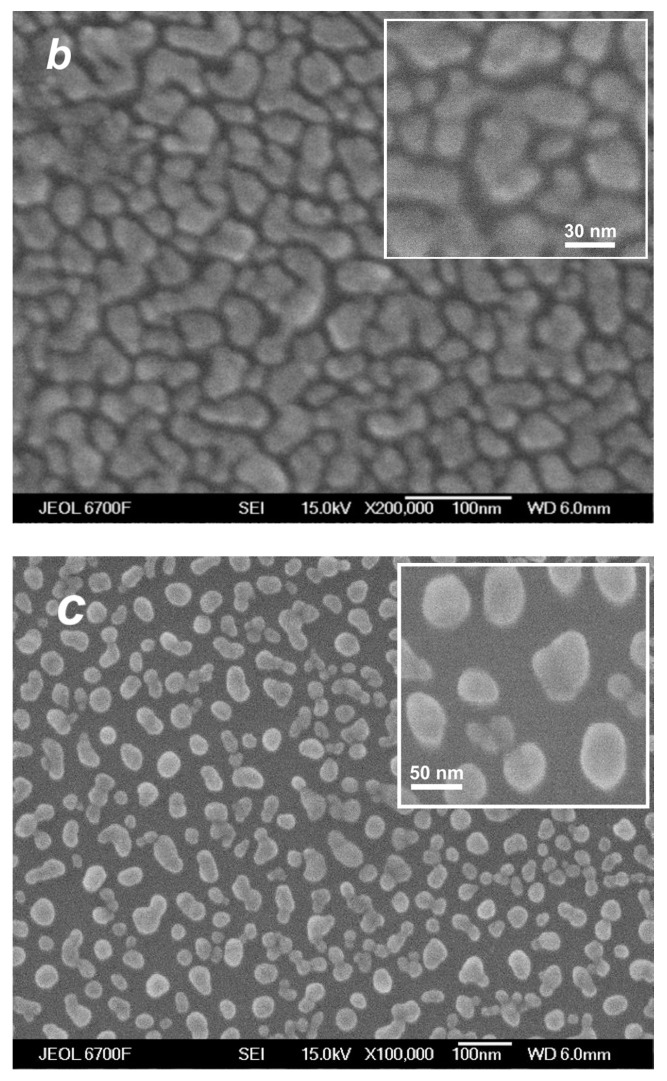
SEM images of silver films of the same mass thickness of ~12 nm deposited on silicon substrates in atomic (**a**) and cluster (**b**,**c**) VGJD regimes. The He flow rate and deposition time were 0 sccm and 150 s for (**a**), 500 sccm and 10 s for (**b**), and 100 sccm and 28 s for (**c**). The source temperature was 1373 K, and the substrates were located 310 mm from the nozzle exit. The insets show at a higher magnification the film nanoparticles obtained in the cluster regimes.

The images shown in Figure 10 do not provide direct information on the influence of small Ag clusters in the deposited flow on the film structure, even if they were obtained in cluster and non-cluster regimes, since the thicknesses of the produced films were different and the film morphology was governed mainly by the amount of the deposited material [45,80]. To reveal the role of the jet Ag_2_ dimers in the film formation process, we adjusted correspondingly the deposition time in different regimes and compared films of identical thicknesses produced with and without silver dimers in the jet. Figure 13 shows SEM images of such films, 12 nm thick each. All the films were nanostructured, although the morphologies were different. The islands of the films produced in the presence of Ag_2_ dimers in the jet (Figure 13b,c) were considerably larger, and their surface densities were lower compared to the film obtained in the atomic VGJD regime (Figure 13a). Therewith, at a lower flow rate of the carrier gas ($Q_{\mathrm{He}}$ = 100 sccm), when the dimer fraction in the jet was smaller (Figure 7c) the obtained nanoparticles were lower in concentration and slightly larger (average size of <*D*> = 30 nm, Figure 13c) than those obtained at $Q_{\mathrm{He}}$ = 500 sccm (<*D*> = 27 nm, Figure 13b). We believe that the deposited dimers, being the critical nuclei at the used room-temperature substrates [78], served as nucleation centers, thus promoting the formation of individual nanoparticles at the substrate surface [22,80]. Therefore, the first step of the island film formation, the nucleation of stable clusters at the substrate, was not needed since they were provided with the flow and efficiently collected the diffusing Ag adatoms during the growth stage [81]. The size of the produced surface nanostructures could, thus, be controlled by varying the cluster fraction in the deposited silver vapor jet.

We should mention here that the increase in the helium flow, apart from promoting Ag cluster generation in the jet, simultaneously changed two other parameters of the flow that could affect the film morphology. With increasing the $Q_{\mathrm{He}}$ from 0 to 500 sccm, the incident kinetic energy of Ag atoms increased from 0.15 eV to 1.7 eV, which could increase their surface mobility [33]. It should be noted that these kinetic energies were considerably lower than those typically realized in the PLD of metals (10–100 eV [43,80]) and were not sufficient to introduce structural defects in the surface. Another parameter, the deposited particle flux, varied significantly with $Q_{\mathrm{He}}$ and also played an important role in the film growth [33,80]. More studies are needed to separate the effects of different factors and to reveal unambiguously the role of the deposited dimers in film structure formation.

## 5. Conclusions

The expansion of a supersonic free jet of silver vapor from a nozzle to a vacuum at a relatively low source temperature of 1373 K was investigated experimentally and theoretically in order to reveal the possibility of the generation of small silver clusters in the jet and to optimize the jet method of thin-film deposition. The main attention was paid to the effect of the helium carrier gas on the jet composition and expansion dynamics. The concentration and velocity of silver particles at the jet axis were measured using time-of-flight mass spectrometry (MS) as a function of helium flow. The general flow field of the silver vapor expansion and formation of clusters in the jet were analyzed with the direct-simulation Monte Carlo (DSMC) method. The pure silver jet without the carrier gas was nearly collisionless under the considered conditions, and its expansion was well-described by the free molecular gas solution. No clusters were found in this regime. Introducing the carrier gas into the source resulted in dramatic changes in the Ag jet, which became faster, denser, and more forward-directed, and the expansion regime became collisional. All these changes were favorable for cluster formation and film deposition. At fairly high helium flows ($Q_{\mathrm{He}}$ > 100 sccm), silver Ag_2_ dimers were observed in the jet, both in the experiment and the simulations, with a mole fraction reaching 0.1% at $Q_{\mathrm{He}}$ > 400 sccm, although a formal application of Hagena’s scaling law did not predict the formation of small silver clusters under these nozzle conditions. The terminal velocities of silver atoms and dimers were found to be nearly identical (although there was a considerable velocity slip between Ag and He). This indicated that the observed clusters were likely formed due to the gas-phase condensation process in the expanding jet and were not ejected from the nozzle walls. The mass spectrometric and DSMC results of both the jet expansion dynamics and cluster formation were in good agreement. We should note, however, that, to achieve an agreement with the experiment on the cluster abundances, we used rate constants for the reaction dimerization (Equations (3)–(6)) different than those estimated based on the simple models of three-particle collisions of hard spheres. This indicated that the gas-phase aggregation reactions in silver vapor involved an orbiting motion of the colliding particles, with the formation of a metastable dimer with a lifetime of the order of 0.1–1 ns.

A high potential of supersonic Ag-He jets for the deposition of high-quality thin silver films, both nanostructured and continuous, was demonstrated. The obtained deposition rates exceeded those of the traditional techniques, molecular beam epitaxy and pulsed-laser deposition, by at least two orders of magnitude. When deposited on a substrate, the small silver clusters formed in the jet appeared to serve as nucleation centers for nanoparticle formation and, thus, allowed for controlling the size of the produced surface nanostructures by adjusting the cluster abundance in the jet.

We believe that VGJD-produced nanostructured films with their high purity, outstanding uniformity over large deposition areas, and controllable island size can be efficiently used in various applications. In addition to those mentioned in the Introduction, we can indicate here specific applications for which the obtained silver nanostructures appear to be particularly attractive. Ag nanoparticles are considered as effective antimicrobial inhibitors [82], e.g., in medical devices and air-conditioning systems. Among different catalytic applications, the use of silver nanoparticles for the selective oxidation of styrene is particularly promising [83]. We recently demonstrated that VGJD-fabricated silver nanostructures could be efficient as SERS substrates [22].

## Data Availability

Data are contained within the article.
